# Spatiotemporal variation in the competitive environment, with implications for how climate change may affect a species with parental care

**DOI:** 10.1002/ece3.9972

**Published:** 2023-04-10

**Authors:** Ahva L. Potticary, Hans W. Otto, Joseph V. McHugh, Allen J. Moore

**Affiliations:** ^1^ Department of Entomology University of Georgia Athens Georgia 30606 USA; ^2^ Department of Ecology and Evolutionary Biology University of Arizona Tucson Arizona 85721 USA

**Keywords:** burying beetle, latitudinal, longitudinal, niche partitioning

## Abstract

Burying beetles of the genus *Nicrophorus* have become a model for studying the evolution of complex parental care in laboratory studies. *Nicrophorus* species depend on small vertebrate carcasses to breed, which they process and provision to their begging offspring. However, vertebrate carcasses are highly sought after by a wide variety of species and so competition is expected to be critical to the evolution of parental care. Despite this, the competitive environment for *Nicrophorus* is rarely characterized in the wild and remains a missing factor in laboratory studies. Here, we performed a systematic sampling of *Nicrophorus orbicollis* living near the southern extent of their range at Whitehall Forest in Clarke County, Georgia, USA. We determined the density of *N. orbicollis* and other necrophilous species that may affect the availability of this breeding resource through interference or exploitation competition. In addition, we characterize body size, a key trait involved in competitive ability, for all *Nicrophorus* species at Whitehall Forest throughout the season. Finally, we compare our findings to other published natural history data for Nicrophorines. We document a significantly longer active season than was observed 20 years previously at Whitehall Forest for both *N. orbicollis* and *Nicrophorus tomentosus*, potentially due to climate change. As expected, the adult body size of *N. orbicollis* was larger than *N. tomentosus*, the only other *Nicrophorus* species that was captured in 2022 at Whitehall Forest. The other most prevalent insects captured included species in the families Staphylinidae, Histeridae, Scarabaeidae, and Elateridae, which may act as competitors or predators of *Nicrophorus* young. Together, our results indicate significant variation in intra‐ and interspecific competition relative to populations within the *N. orbicollis* range. These findings suggest that the competitive environment shows extensive spatiotemporal variation, providing the basis to make predictions for how ecology may influence parenting in this species.

## Graphical abstract

The burying beetle *N. orbicollis* competes for small vertebrate carcasses that it uses for breeding, and upon securing a carcass, shows complex parental care to its offspring. Thus, competition is important for care, and yet the competitive environment is rarely characterized in the wild. We document extensive spatiotemporal variation in the necrophilous community both within and across populations of *N. orbicollis* and potential impacts of climate change on the competitive environment.

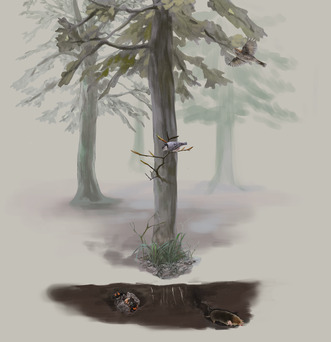

## 1 INTRODUCTION

Burying beetles of the genus *Nicrophorus* (Silphidae) have long intrigued evolutionary biologists due to their complex parental care behavior (Eggert & Müller, 1997; Pukowski, 1933; Scott, 1998). Broadly, parental care is expected to evolve to ameliorate harsh environments for offspring (Wilson, 1975), and variation in parental care can be driven by ecological contexts that change the costs and benefits of allocating effort to current and future reproductive opportunities (Richardson et al., 2020; Stearns, 1989). The costs and benefits of parental care, and what strategies are pursued, are determined in part by competition for mates and breeding resources (Richardson et al., 2020). Moreover, parental care in *Nicrophorus* is known to influence development of offspring body size, a key trait essential for competition over breeding resources (Hopwood et al., 2014; Jarrett et al., 2017; Lee et al., 2014; Otronen, 1988). The active season of burying beetles can encompass multiple generations that experience different competitive environments depending on habitat and when individuals reach adulthood (Anderson, 1982; DeMoss, 1968; Eggert & Müller, 1997; Meierhofer et al., 1999; Scott & Trainello, 1990, 1998). Thus, the costs and benefits of parental care depend on ecological context and variation in parental care directly influences offspring traits that are related to the ability to win access to resources in the next generation. For this reason, there is expected to be reciprocal feedback between the competitive environment experienced by parents, impacts on offspring development, and how this influences the competitive environment of offspring. This feedback will depend critically on the sources of competition, the competitors, and the time of year that a beetle reaches sexual maturity. Thus, understanding the evolution of parental care in this system requires an understanding of how competition and ecological context vary over an active season in wild populations.

**FIGURE 1:**
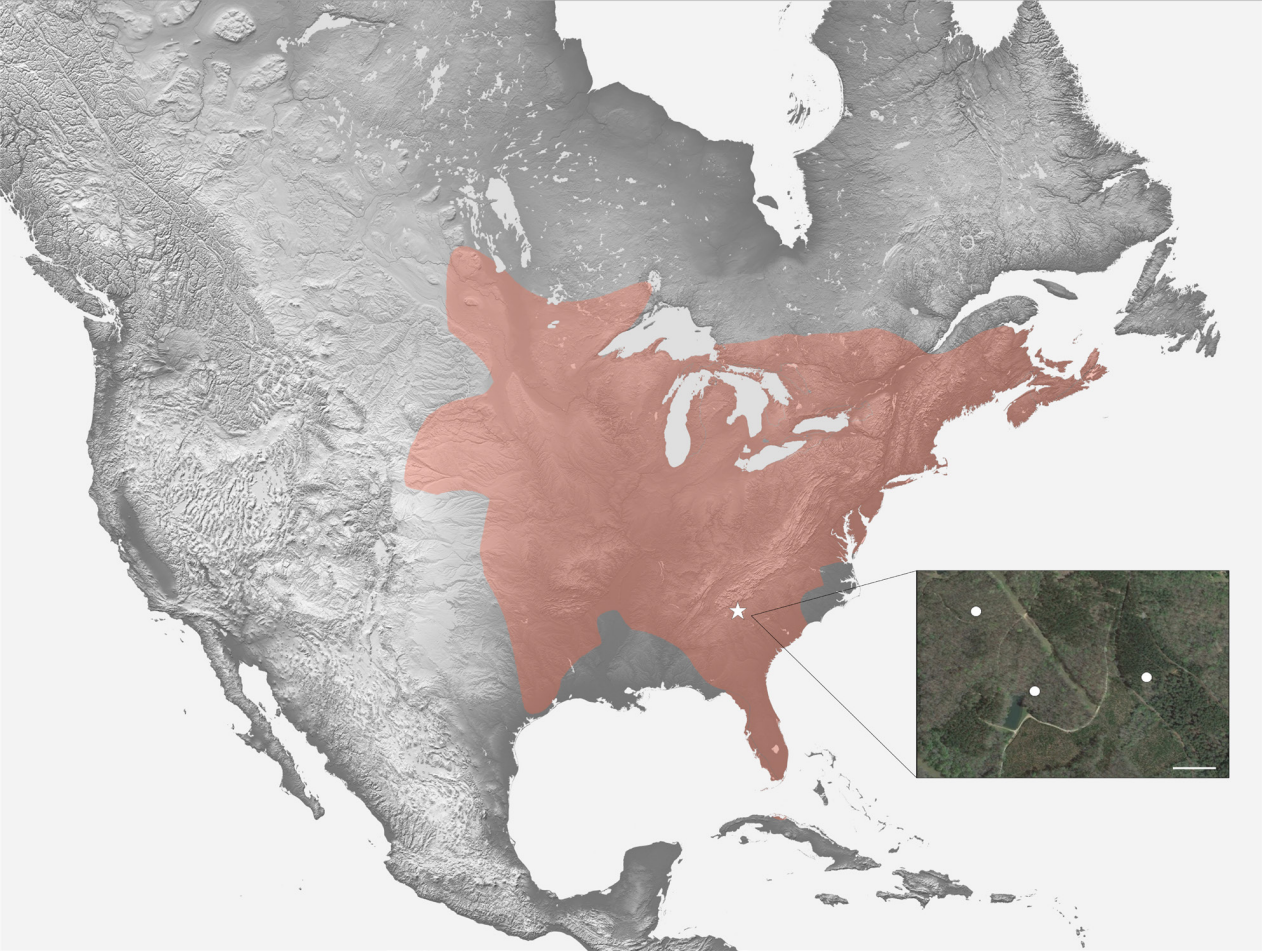
Range of *N. orbicollis* and location of study sites. Map derived from verified iNaturalist sightings indicating the general range of *N. orbicollis* (light orange overlay). Inset indicates relative location of study area within the *N. orbicollis* range and distribution of trap lines (white circles) within Whitehall Forest. Scale bar indicates 150 m.

Here, we characterize the competitive environment and traits related to competition in *Nicrophorus orbicollis* across its active season in the southern portion of their range (Figure 1). *N. orbicollis*, like most Nicrophorinae, require small vertebrate carcasses to breed, which are an ephemeral and highly sought‐after resource. Body size is a key competitive trait for *Nicrophorus*, as larger beetles usually win contests for carcasses (Otronen, 1988; Robertson, 1993), and parental care itself has a strong influence on offspring development and adult body size (Hopwood et al., 2014; Jarrett et al., 2017). Parental care in *N. orbicollis* is thought to have evolved to buffer offspring from competition, predation by scavengers, and decomposition of the carcass by bacteria and fungi (Eggert & Müller, 1997; Scott, 1998). Once beetles have found a carcass, they strip the exterior (e.g., fur or feathers), bury, and maintain the carcass with an elaborate cocktail of secretions that minimize decomposition (Arce et al., 2012; Eggert & Müller, 1997; Scott, 1998). Following the hatch of larvae, parents then regurgitate predigested carrion to their begging larvae (Milne & Milne, 1976; Pukowski, 1933).

Burying beetle parents actively defend their carcass from intra‐ and interspecific intruders. These interactions can be influenced by temperature (Wilson et al., 1984), and parental care itself may be influenced by temperature (Benowitz et al., 2019; Meierhofer et al., 1999). Temperature affects seasonal transitions between overwintering behavior and insect species that use carrion show varying sensitivities to temperature and humidity, which likely influence the competitive environment (Wettlaufer et al., 2023; Wilson et al., 1984). *Nicrophorus* species show temporal niche partitioning during both circadian and seasonal cycles that are likely supported physiologically by differences in temperature tolerance (Anderson, 1982; Keller et al., 2019; Wettlaufer et al., 2023). Moreover, burial and prevention of decomposition may help to prevent discovery of the carcass by scavengers and competitors (Pagh et al., 2015; Robertson, 1993; Trumbo & Sikes, 2021), particularly because decomposition is strongly affected by temperature. Outcomes of competition and population dynamics thus reflect a combination of species presence and their activity levels at different temperatures. For this reason, we collected temperature, phenological, and competition data to determine how the competitive environment changes over the active season.

## 2 MATERIALS AND METHODS

### 2.1 Range map and study area

During the spring—fall of 2022, we conducted a survey of *N. orbicollis* abundance and sympatric necrophilous insects at Whitehall Forest in Clarke County, Georgia, USA, located in the southern portion of the *N. orbicollis* range (Figure 1; 33.8848°N, 83.3577°W). There are no recent range maps of *N. orbicollis* in the literature (but see Anderson & Peck, 1985); thus, we constructed an approximate range map for *N. orbicollis* using verified “research‐grade” iNaturalist (www.inaturalist.org) observations occurring from 2000 to 2023, downloaded on February 23, 2023, from the iNaturalist site (*n* = 2686 observations) to produce a map of verified *N. orbicollis* observations. All outliers, defined as individual points more than 100 miles from other observations, were assessed visually and by reviewing photographic and location evidence in the observation description. Based solely on these data we produced a map in Adobe® Photoshop (v. 20.0.8; https://adobe.com) to show the approximate distribution of *N. orbicollis*, although it is likely that *N. orbicollis* occupy habitat outside the scope of our range map. We drew the boundaries by hand following a convex hull approach. The *N. orbicollis* range was overlaid on a topographic map generated by NASA's Shuttle Radar Topography Mission in 2000 (https://photojournal.jpl.nasa.gov/catalog/PIA03377) and was rendered to grayscale in Adobe Photoshop to enable clearer visualization of their range.

Whitehall Forest is a discontinuous stretch of forest managed by the University of Georgia Warnell School of Forestry and covers approximately 840 acres of fragmented forest surrounded by residential development in the Southern Outer Piedmont. The forest is comprised primarily of natural pine, planted pine, upland hardwood, and bottomland hardwood. Within our specific trapping areas, the predominant tree species included red maple (*Acer rubrum*), American hornbeam (*Carpinus caroliniana*), flowering dogwood (*Cornus florida*), North American beech (*Fagus grandifolia*), yellow poplar (*Liriodendron tulipifera*), sweetgum (*Liquidambar styraciflua*), shortleaf pine (*Pinus echinata*), southern red oak (*Quercus falcata*), post oak (*Quercus stellata*), and white elm (*Ulmus americana*). Understory was minimal, and the forest was primarily characterized by large, well‐dispersed trees with deep leaf litter. The study sites are adjacent to habitat including brushy pastures and grassy fields, and much of Whitehall Forest is interspersed with areas of maintained grassland or areas of intermittent prescribed burns (Figure 1 inset). Based on maps generated from the USDA Soil Survey, soil in the study area is primarily comprised of sandy clay loam, loamy sandy, and alluvial land (Appendix S1), similar to other sites supporting *N. orbicollis* (DeMoss, 1968). Humus content was also high due to a large amount of leaf litter across the entirety of the study site.

### 2.2 Trapping and life history of *Nicrophorus orbicollis* in Georgia

Beetles were trapped using hanging Japanese beetle traps (hereafter, traps; item no. 227723, Trécé Inc., Adair, OK, USA) baited with one‐inch square cubes of salmon along three transect lines of five traps each (Figure 1 inset). Traps were hung approximately 1 m off the ground in a tree that shaded the trap. We started trapping on March 1 and defined the onset of the *N. orbicollis* active season as when the first *N. orbicollis* was captured. We determined the end of the active season as two consecutive trapping events with no *N. orbicollis* captures. One transect was adjacent to a small pond and perennial stream, while the other two transects did not have permanent water. *N. orbicollis* is primarily found in woodland areas (Anderson, 1982); therefore, all traps were placed within woodland habitat. Traps were checked every 6–9 days from March 1 to December 15, except for a few instances where traps could not be checked at this frequency and the bait was not replaced for 14 days. In traps, the salmon bait was contained in a small plastic cup that greatly reduced desiccation and insect scavenger consumption of the bait. For this reason, even though bait was not replaced weekly on several occasions, it was still present and able to attract beetles, as adults of *Nicrophorus* species are known to preferentially eat late stage decomposing meat (e.g., Dekeirsschieter et al., 2011; Rodriguez & Bass, 1983; von Hoermann et al., 2013). We thus calculated the number of beetles captured as the number per trap day to account for variation in when traps were checked. We further noted any teneral beetles (recently eclosed adults that have not yet become fully melanized and can be identified because they are light brown instead of black), as they provide an indication of recent breeding activity.

We visualized the length of the activity period of *N. orbicollis* over the field season with data from similar studies both temporally within Whitehall Forest, using data collected in the study area in 2002 (Ulyshen & Hanula, 2003), and spatially, by using data from the central (Kentucky; DeMoss, 1968) and northern (Ontario; Anderson, 1982) portions of the *N. orbicollis* range. While all three studies used different trapping methods, e.g., Anderson (1982) used pitfall traps baited with carrion, their data are roughly comparable to data collected for this study as they both show seasonal activity of *N. orbicollis*, as well as periods of peak activity. Data were readily available in a tabular form in DeMoss (1968) but raw data were collected from Figure 8 in Anderson (1982) and Figure 1 in Ulyshen and Hanula (2003) using the online platform PlotDigitizer (https://plotdigitizer.com/). All three studies collected data weekly, although there were gaps in the collection times across studies. To allow for visualization across studies that differed in the date that traps were checked, we assigned a single date to trap dates that were most closely aligned (i.e., three or fewer days). To account for gaps in data collection at Whitehall Forest relative to other sites, we summed the number of beetles collected at other sites over the same time periods when traps in Georgia were not checked.

### 2.3 Characterization of the competitive environment

We characterized the competitive environment for *N. orbicollis* by identifying all species of insects attracted to carrion in traps. Furthermore, we identified all individuals captured to genus and to species, if possible (Table 1), apart from those that appeared two or fewer times, as these likely reflect incidental bycatch. A voucher specimen of each species was collected and pinned to allow for identification, and we identified each species using multiple taxonomical resources (Aalbu et al., 2002; Ball et al., 2000; Ciegler, 2000, 2014; Harpootlian & Morse, 2001; Johnson, 2002; Kovarik & Caterino, 2000; Mathison, 2021; Pollock, 2002; Ratcliffe et al., 2002). We measured pronotum length of all *Nicrophorus* captured to the nearest 0.01 mm using digital calipers as a proxy for body size and identified the sex of each beetle. A small subset of the *N. orbicollis* captured were retained (*n* = 144) to augment our laboratory population. Beetles sometimes escaped after sexing but prior to measurement (*N* = 3) or were too damaged or desiccated, in which case, they were identified to species but no sex or pronotum length data were collected (*N* = 77). Since burying beetles can travel long distances to find carrion, e.g., *Nicrophorus americanus* can travel 7.24 km in a single night (Jurzenski et al., 2011), the retention of a small number of individuals is unlikely to significantly impact local populations or results of trapping efforts. We did not retain more than 10 individuals in a week as a precaution. Also, it is likely that some individuals were recaptured. Despite this, our capture methods were intended to demonstrate the number of individuals competing for carrion at any given time rather than provide total numbers of individuals in the study site and are thus still appropriate and representative of changes in the competitive environment.

**TABLE 1:** Species captured in carrion traps across *N. orbicollis* active season.

Family	Species name	Total captured (female/male)	Capture dates
Silphidae	*Nicrophorus orbicollis*	703 (373/253)	March 24–November 18
	*Nicrophorus tomentosus*	53 (38/15)	May 6–December 8
	*Necrophila americana*	944	April 29–September 2
	*Oiceoptoma inaequale*	464	April 1–June 21
	*Oiceoptoma noveboracense*	39	May 6–June 21
	*Necrodes surinamensis*	1	November 22
Elateridae	*Gambrinus griseus*	20	May 6–September 2
	*Melanotus similis*		
	*Orthostethus infuscatus*	54	June 9–July 14
Histeridae	*Euspilotus* sp.	1	06‐May
	*Unknown* spp.	86	May 6–September 2
Scarabaeidae	*Deltochilum gibbosum*	39	May 6–October 6
	*Serica* sp.	32	April 1–26

*Note*: Species for which at least ten were captured in carrion traps. Where possible, individuals were identified to species, although several were only identified to genus or family. For *Nicrophorus* species, individuals were also identified to sex, as described below the total capture numbers. Capture dates reflect the first and last dates individuals were captured for each.

We collected ambient temperature and precipitation data from the University of Georgia Weather Network (College of Agricultural and Environmental Sciences, University of Georgia), using the Watkinsville‐HORT Weather Station, located 5.15 km from the nearest trapping location.

*N. orbicollis* are known to survive overwintering at soil depths between 5 cm and 105 cm in more northern portions of their range (Hoback & Conley, 2015). For this reason, we measured soil temperature by placing Thermochron® iButton temperature loggers (©Maxim Integrated Products, Inc.) approximately 10–12 cm underground at the start of each transect line in October to determine the soil temperature associated with *Nicrophorus* diapause in our study area.

### 2.4 Statistical analyses

We analyzed pronotum length of individuals by sex and by species using two‐way ANOVA with sex and species as factors. We performed all statistical analyses using JMP Pro (v. 16.0.0, http://jmp.com) and produced figures in SigmaPlot (v. 14.5, http://www.sigmaplot.co.uk). Results are means ± SE unless otherwise noted.

## 3 RESULTS

### 3.1 Life history of *N. orbicollis* in Georgia

**FIGURE 2:**
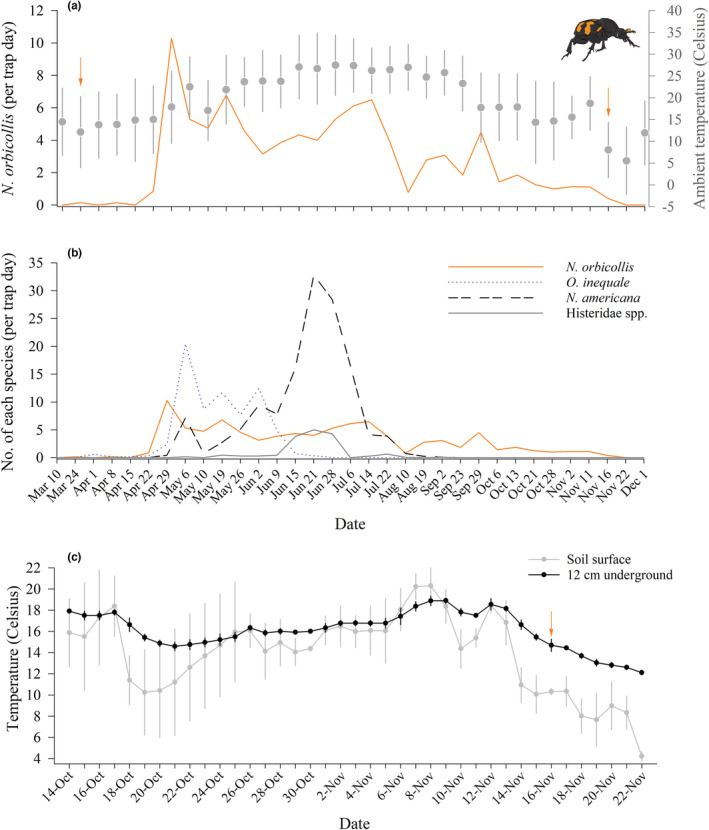
Activity of *N. orbicollis* relative to environmental context. (*a*) Number of *N. orbicollis* captured per trap day across the active season (orange line). Orange arrows indicate dates when the first or last *N. orbicollis* were captured for the season. Gray y‐axis indicates mean ambient temperature (gray circle and error bars) from nearby weather station. (*b*) Number of each species per trap day for the three most abundant interspecific competitors (*Oiceoptoma inaequale*, *Necrophila americana*, and Histeridae spp.) for carrion relative to *N. orbicollis* captures. X‐axis is the same for both *A* and *B*. (*c*) Temperature at soil surface (gray line and gray circles) and 12 cm underground (black line and black circles) in the study area. Orange arrow indicates when the last *N. orbicollis* was captured. Note that temperature data from the study area are only available for October 14–November 22, 2022. Error bars ± SD.

We observed the first *N. orbicollis* activity in traps on 24 March (1 adult male, 1 adult female; Figure 2a) and captured the last *N. orbicollis* on November 16, roughly corresponding to when soil temperatures 12 cm underground fell below 14°C (Figure 2c). We captured four teneral *N. orbicollis* adults on 28 June. Developmental periods for *N. orbicollis* in the laboratory are approximately 45 days from egg to adult (Potticary et al., in review). Thus, these data suggest that the parents of these individuals laid eggs between mid‐ and late‐May.

**FIGURE 3:**
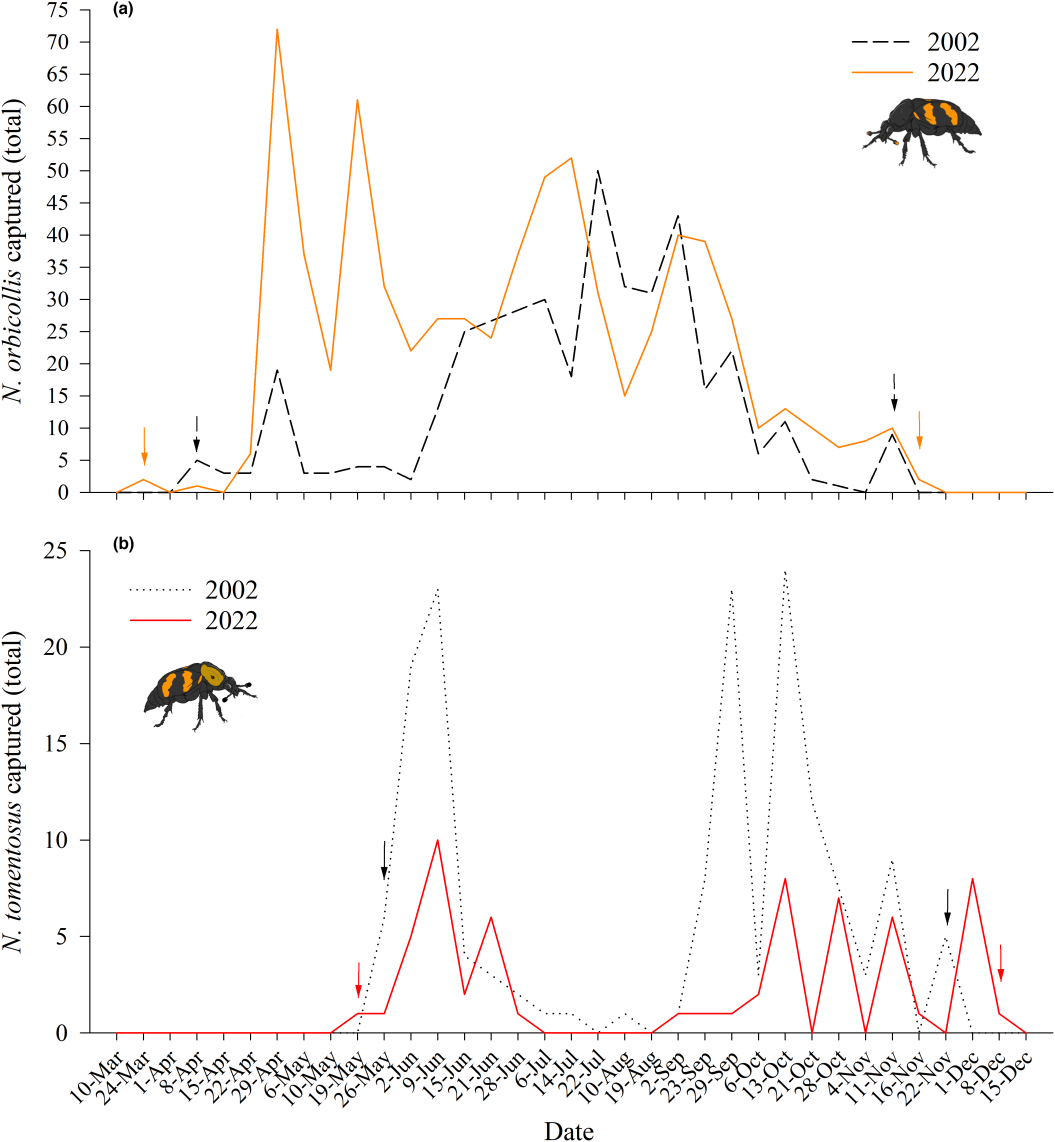
*Nicrophorus* active season has extended over 20 years. (*a*) *N. orbicollis* total captures at Whitehall Forest for 2002 (dashed black line) and 2022 (solid orange line). Black arrows indicate dates that *N. orbicollis* were first or last captured in 2002, while orange arrows indicate dates that *N. orbicollis* were first or last captured in 2022. (*b*) *N. tomentosus* total captures at Whitehall Forest for 2002 (dotted black line) and 2022 (solid red line). Black arrows indicate dates that *N. tomentosus* were first or last captured in 2002, while red arrows indicate dates that *N. tomentosus* were first or last captured in 2022. Note that scaling of y‐axes for *A* and *B* differ. All 2002 data were acquired from Ulyshen and Hanula (2003).

**FIGURE 4:**
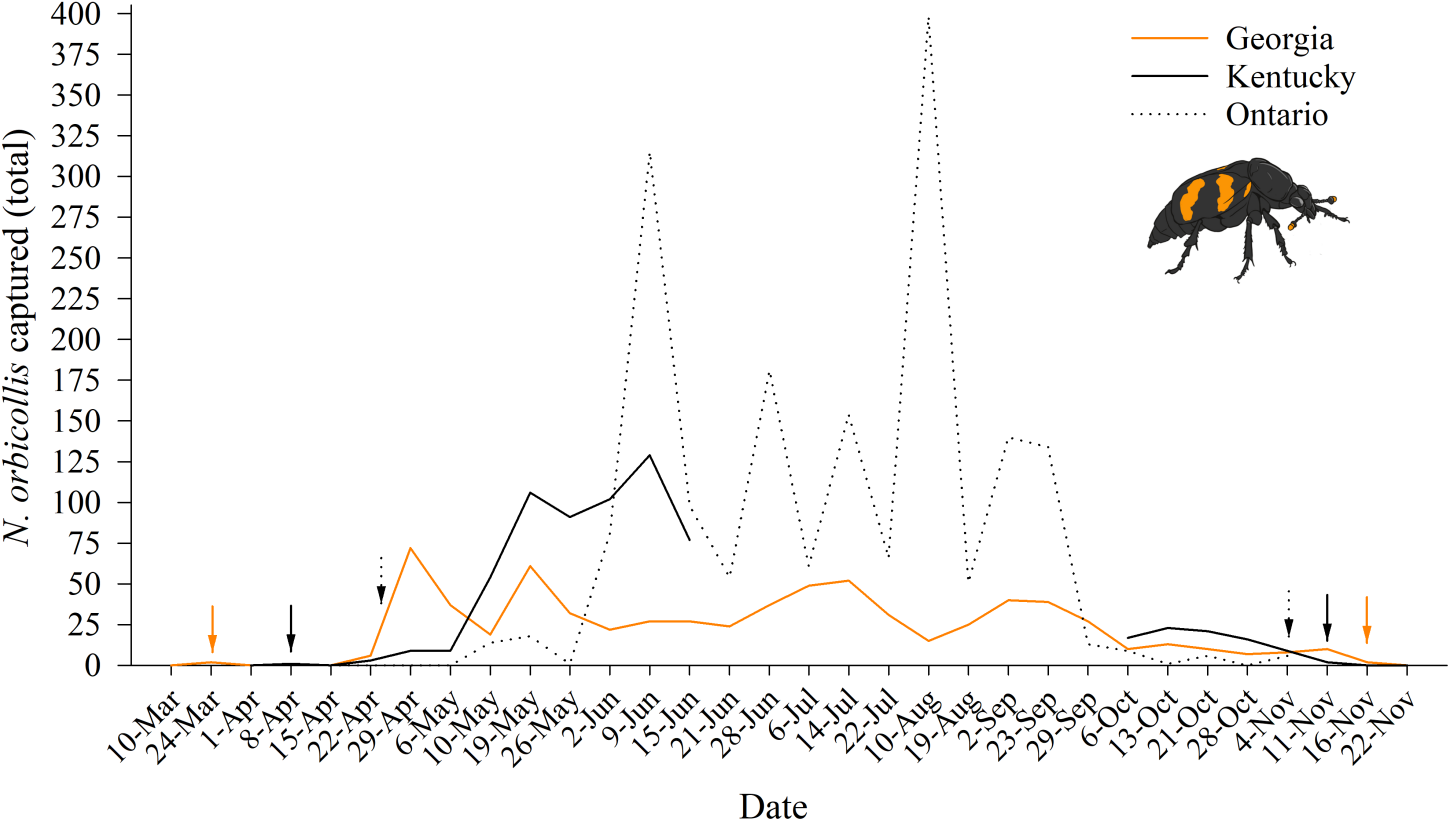
*N. orbicollis* active season longer in more southern areas. Weekly total *N. orbicollis* captures at Whitehall Forest, GA (solid orange line), in Kentucky (solid black line), and Ontario (dotted black line). Orange arrows indicate first and last *N. orbicollis* captures in Georgia, while solid black and dotted arrows indicate the same information for Kentucky (DeMoss, 1968) and Ontario (Anderson, 1982), respectively.

We observed an extension in the active season of *Nicrophorus* species in Whitehall Forest relative to research conducted in the same area in 2002 (Figure 3). *Nicrophorus orbicollis* emerged 2 weeks earlier and entered diapause nearly a week later, an approximately three‐week extension of the active season over 20 years (Figure 3a). We observed a similar pattern in *N. tomentosus*, which emerged a week earlier and entered diapause 2 weeks later than in 2002 (Figure 3b). Across latitudes, *N. orbicollis* in more northern populations emerge later and enter diapause sooner than in Georgia, showing higher and more condensed bursts of activity (Figure 4). Together, these data support the idea that the length of the *N. orbicollis* active season tracks temperature both within and across populations.

### 3.2 Characterization of the inter‐ and intraspecific competitive environment

A total of 703 *N. orbicollis* were captured over the season, with the number of females (*N* = 373) exceeding the numbers of males (*N* = 253; Table 1). A similar pattern was observed with *N. tomentosus*; a total of 53 of *N. tomentosus* were captured over the active season, with the number of females (*N* = 38) exceeding the number of males (*N* = 15). The most common non‐*Nicrophorus* species captured in carrion traps were *Necrophila americana*, *Oiceoptoma inaequale*, and Histeridae spp., although multiple potentially necrophilous species were captured (Table 1). The number of individuals of each species captured appears to coincide with a decrease in *N. orbicollis* captures and warmer ambient temperatures (Figure 2b). Peak periods of *N. tomentosus* captures seem to be disjunct from peak periods of *N. orbicollis* captures (Figure 3). The active season of *N. orbicollis* appears to be influenced by latitude, likely due to differences in temperature, with more northern populations having a shorter active season than those farther south (Figure 4).

**FIGURE 5:**
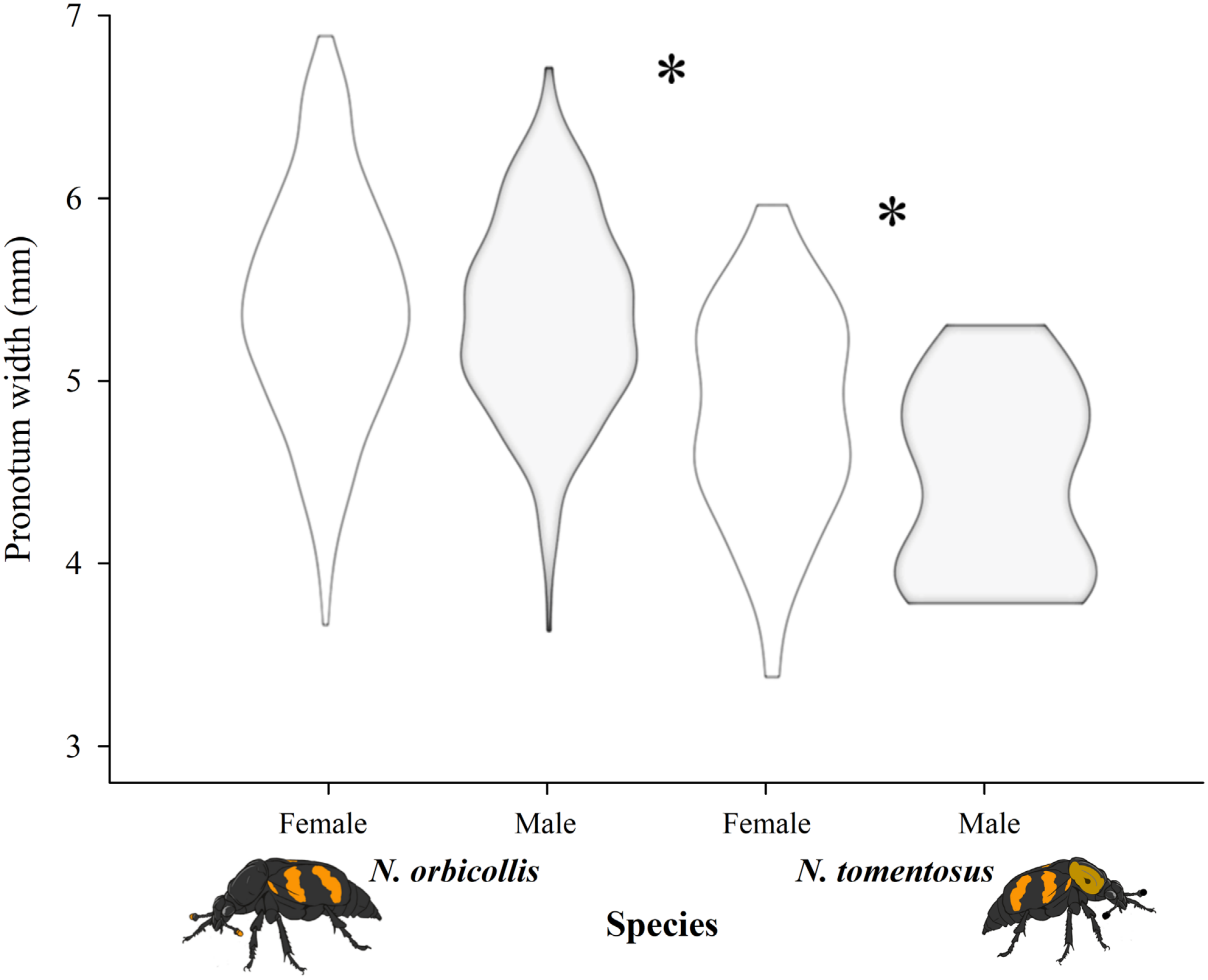
Variation in pronotum size of *Nicrophorus* at Whitehall Forest. Pronotum length of female and male *N. orbicollis* and *N. tomentosus*. While female (white violin) and male (gray violin) *N. orbicollis* do not differ in pronotum length, *N. orbicollis* is larger than *N. tomentosus*, and *N. tomentosus* females are larger than *N. tomentosus* males. Asterisks indicate a statistically significant difference between groups (*p* < .05).

Pronotum length differed between *Nicrophorus* species and sexes in *N. tomentosus* (*F*_3,673_ = 22.0549, *p* < .0001; Figure 5). *N. orbicollis* was larger than *N. tomentosus* (*F*_1,673_ = 62.083, *p* < .0001), and there was an effect of sex on pronotum length (*F*_1,673_ = 5.240, *p* = .0224), but there was no interaction between sex and species on pronotum length (*F*_1,673_ = 2.873, *p* = .091). Pronotum length of female *N. orbicollis* ranged from 3.69 to 6.9 mm (mean 5.43 ± 0.03 mm, *N* = 371), while pronotum length of male *N. orbicollis* ranged from 3.65 to 6.73 mm (mean 5.37 ± 0.04 mm, *N* = 252), but there was no difference in the size of male and female *N. orbicollis*. Pronotum length of *N. tomentosus* females range from 3.4 to 5.96 mm (mean 4.83 ± 0.09 mm, *N* = 37), and male *N. tomentosus* body size ranged from 3.8 to 5.3 mm (mean 4.45 ± 0.15 mm, *N* = 15). Female *N. tomentosus* were generally larger than male *N. tomentosus*, but there was a large discrepancy in the sample size between males and females.

## 4 DISCUSSION

The costs and benefits of parental care are expected to depend on access to resources and for this reason, the competitive environment experienced by parents is expected to influence the evolution of parental care strategies. We characterized the competitive environment across the active season for *N. orbicollis* breeding in a population located in southern portion of their range and compared these results to similar studies conducted at different temporal and spatial locations across their range. In Georgia, *N. orbicollis* densities were highest in late April–late May and mid‐June to early August, demonstrating a different peak in activity than in more northern portions of the *N. orbicollis* range. We captured multiple interspecific competitors for carrion and potential predators of larvae; some of these species co‐occur with *N. orbicollis* throughout its range and show extreme variation in phenology of activity over the season. Of particular interest, we document that *Nicrophorus* species currently emerge earlier and enter diapause later in Whitehall Forest than 20 years ago.

The total number of *N. orbicollis* captured in this study was over double the number of individuals captured in Whitehall Forest in 2002, while there was nearly a 2/3 decrease in the number of *N. tomentosus* captured in 2022 relative to 2002 (Figure 3; Ulyshen & Hanula, 2003). While there are methodological differences between these studies—e.g., Ulyshen and Hanula (2003) killed and collected all individuals and baited traps with chicken—there are several lines of evidence suggesting that these differences reflect increases in *N. orbicollis* density. First, there is no evidence suggesting that either burying beetle species prefers chicken to salmon or that the number of traps affected capture success, as similar numbers of *N. orbicollis* were captured for the last half of our field seasons. Second, our laboratory maintained the same trap lines in 2021 and collected 630 *N. orbicollis* to establish a laboratory colony, which also surpasses numbers captured in 2002. The significant decrease in the number of *N. tomentosus* documented between the two studies may also indirectly support that *N. orbicollis* populations are increasing. *N. tomentosus* are generally competitively subordinate to *N. orbicollis* (Schrempf et al., 2021), such that there is temporal niche partitioning between periods of activity of *N. orbicollis* and *N. tomentosus* (Anderson, 1982; Keller et al., 2019; Wilson et al., 1984). In accordance with previous research, we also found that *N. tomentosus* are more abundant in traps when *N. orbicollis* captures are low. An increase in *N. orbicollis* populations could provide one possible explanation for the drastic decrease in *N. tomentosus* at our study site. We suggest it would be valuable to use mark–recapture methods to derive estimates of population‐level change over time in future research.

We captured more females than males of both *Nicrophorus* species. Across *Nicrophorus* species, some studies have documented roughly equal sex ratios in the wild (Anderson, 1982; Milne & Milne, 1976; Otronen, 1988), while others have also noted higher captures rates of females than males (Collard et al., 2021; Conley, 1982; Sikes, 1996; Trumbo, 1990). However, this is not necessarily indicative of differences in secondary sex ratios. Variation in captures may reflect differences in attraction to different types of carrion, such as state of decomposition, size, and species of carrion, as this may attract beetles at different reproductive stages (Delclos et al., 2021; Otronen, 1988; Wilson & Knollenberg, 1984). Males may not be as attracted to carcasses as females as they can “call” for females off a carcass and so males may split their time between searching for carcasses and calling for females (Beeler et al., 1999; Eggert & Müller, 1997; Müller & Eggert, 1987). Moreover, our laboratory colony is derived from beetles at Whitehall Forest, and sex ratios of beetles that survive to adulthood are almost exactly 1:1 (Potticary et al., in review). This provides indirect evidence that the discrepancy in captures between the sexes may reflect behavioral differences in response to variation in carrion, although future research would be needed to address this effect.

On a short timescale, the number of *Nicrophorus* that we captured likely reflect a combination of total individuals, temperature, food, and how many individuals are underground breeding and thus not searching for carrion. On an evolutionary timescale, we would predict that peak *Nicrophorus* abundance should reflect an evolved response to ideal breeding conditions. In Georgia, April and May were periods of peak *N. orbicollis* capture, which correspond roughly to avian breeding in Whitehall Forest, and patterns are similar across latitudes. *Nicrophorus orbicollis* depends on carcasses, which means that vertebrates dying of predation are unlikely to be available for breeding. Nestling and fledgling mortality of avian species due to exposure is high, and adults deposit dead nestlings away from the nest, which may provide a predictable source of carcasses for burying beetles. In addition, small mammal species are often plentiful in spring and early summer in North America (Merritt, 2010), and even though a reliable measure of mortality is difficult to obtain in some species, nonpredator‐related mortality (e.g., food limitation, habitat conditions, and disease) in larger populations could yield more carcass availability in spring. Together, these may provide a predicable source of breeding resources during certain months. However, future research is needed to determine the factors that influence peak activity periods on both local and evolutionary scales across the *N. orbicollis* range.

While *Nicrophorus* are often expected to be the only species that compete via interference competition for breeding carcasses, we documented multiple species that may reduce the availability of carcasses through exploitation competition (e.g., *Necrophila americana*) or that may be able to outcompete *Nicrophorus* due to larger body size (e.g., *Deltochilum gibbosum*). The most common interspecific competitors captured, in order of abundance, included *Necrophila americana*, *Oiceoptoma inequale*, *N. tomentosus*, *Deltochilum gibbosum*, and *Oiceoptoma noveboracense*. Some of these species are common across the range of *N. orbicollis*, albeit at different densities (e.g., Anderson, 1982; Wettlaufer et al., 2021). *N. orbicollis* alter both the number of eggs laid (Scott, 1997) and brood size (Creighton, 2005; Smith et al., 2015) based on assessment of carcass size (Trumbo & Fernandez, 1995). For this reason, even species that reduce the availability of carrion by eating it are expected to influence *Nicrophorus* parental strategies, although future research would be needed to determine to what extent interference or exploitation competition impact parental care of *Nicrophorus*.

We captured multiple species for which it was unclear whether they were acting as competitors or predators of larvae on carcasses, including several species of the beetle families Elateridae and Histeridae. Histerid beetles were one of the most abundant groups that we captured, although only one specimen was identified to genus (*Euspilotus* sp.). Many histerid species are generalist predators that are known to prey upon small arthropods, including the immature and adult stages of other insects, and some histerid species are necrophilous (Correa et al., 2020). Histerid species are found worldwide and an estimated 6% are associated with carrion (Correa et al., 2020). Histeridae species have been captured in conjunction with *Nicrophorus* species in this study and other ecological studies all over the world (Naranjo‐López et al., 2011; Psarev et al., 2020; Shubeck, 1983). Indeed, Naranjo‐López and Navarrete‐Heredia (2011) found that Histeridae species were one of the most abundant groups captured with *Nicrophorus olidus* in Mexico. Despite the widespread overlap of Histeridae and *Nicrophorus* species, there is little known about how hister beetles may impact breeding *Nicrophorus*. The threat of predation to offspring is expected to be a major factor in the evolution of parental care in insects like burying beetles (Scott, 1990, 1998; Suzuki & Nagano, 2006; Tallamy, 1984; Trumbo, 2022), and thus widespread predators should be of importance for behavioral evolution across *Nicrophorus*. Future research should investigate the relationship between *Nicrophorus* and associated histerid and elaterid species and examine any potential impacts on *Nicrophorus* parental care strategies.

The competitive environment an individual experiences, and thus the opportunities for reproduction and parental care, depend on the length of the active season. We documented that *Nicrophorus* species currently emerge earlier and enter diapause later in Whitehall Forest than 20 years ago. While this only constitutes two sampling periods, a difference of nearly three weeks (or about ½ the length of time to develop from egg to adult) in the length of the active season for two different species may indicate that climate change is impacting *Nicrophorus* activity, as warming temperatures have been indicated for Georgia (Frankson et al., 2022). Insect diapause activity can be driven by photoperiod, density, and other factors (reviewed in Gill et al., 2017); however, *Nicrophorus* termination of diapause is likely driven by soil temperature as the beetles are thought to burrow to avoid winter temperatures and are thus unlikely to have access to other cues such as photoperiod (Hoback & Conley, 2015). Moreover, given the broad range for *N. orbicollis*, the most parsimonious explanation is that diapause activity is determined by local temperature cues. This may make *Nicrophorus* species particularly susceptible to changing temperatures resulting from climate change. The mechanisms that govern the duration of diapause and activity periods in *N. orbicollis* are poorly understood and warrant future research, and long‐term data are particularly needed.

*Nicrophorus* breeding in more southern portions of their range, like Georgia, may have multiple generations in a single active season. We found evidence indicating that *N. orbicollis* breeds as early as May at Whitehall Forest. Several teneral adults were observed on 28 June and given the approximately 45‐day egg‐to‐adult developmental times in this species, this indicates an onset of breeding time between mid‐May to late‐May. Anderson (1982) also characterized tenerals in Ontario and they were not detected until August and September. A spike of young adults this close to the end of the active season (concluding in late September), may indicate that in more northern populations there is only a single generation per active season. An earlier onset of breeding in Whitehall Forest, in conjunction with delayed diapause relative to other portions of the range (Anderson, 1982; DeMoss, 1968), indicates that populations in Georgia may have multiple generations within a single season. While the numbers of *Nicrophorus* captured in carrion traps may not reflect the number of breeding adults because even nonreproductive individuals feed on carrion, all *N. orbicollis* that were brought to the lab bred when provided with a suitable carcass, even those caught late in the active season (e.g., November). As the densities of intra‐ and interspecific competitors fluctuate greatly over the active season, early versus late season breeders likely experience drastically different competitive environments, which may have implications for the expression of parental care behavior. Future research should investigate whether the expression of parental care varies in populations with single versus multiple generations per season.

In conclusion, we documented variation in the competitive environment experienced by *N. orbicollis* over space and time. Behavior is a response to a context, and parental care strategies are expected to be particularly influenced by competition in burying beetle species. Thus, an individual burying beetle's experience depends on when they are an adult and the population context of their natal population (Meierhofer et al., 1999). In this study, we document changes in both the length of the active season and the species composition of Whitehall Forest which may be influenced by climate change. Such changes in the length of the active season likely indicate that early and late breeding *Nicrophorus* experience different competitive environments, which may impact the costs and benefits of parental care both across an active season within a site, as well as across populations, particularly since northern populations experience more contracted active seasons. Changing temperatures may further shift the phenology of competitive environment and species composition over time, which may also impact parental care behavior. Future behavioral and ecological research to better understand breeding strategies, activity, distribution, and effects of climate change on *N. orbicollis* is warranted.

## AUTHOR CONTRIBUTIONS

**Ahva Potticary:** Conceptualization (equal); data curation (equal); formal analysis (lead); investigation (equal); methodology (equal); project administration (lead); supervision (lead); visualization (lead); writing – original draft (lead); writing – review and editing (equal). **Hans W. Otto:** Data curation (equal); investigation (equal); methodology (equal); writing – review and editing (equal). **Joseph V. McHugh:** Data curation (equal); investigation (equal); writing – review and editing (equal). **Allen J Moore:** Conceptualization (equal); funding acquisition (lead); methodology (equal); resources (lead); writing – review and editing (equal). ALP, AJM, and HWO conceived of study and developed methods. ALP and HWO collected data. ALP created figures. ALP, HWO, and JVM identified captured species. ALP, HWO, JVM, and AJM all contributed to editing and writing the manuscript. ALP was funded by a USDA cooperative agreement to AJM.

## CONFLICT OF INTEREST STATEMENT

The authors declare no conflict of interest.

## Supporting information


Appendix S1.

Appendix S2.

## Data Availability

Observational data used to build the range map, trapping and morphological data files: Dryad doi: 10.5061/dryad.j6q573njp.
