# Supplementary material for: Spatiotemporal variation in the competitive environment, with implications for how climate change may affect a species with parental care
Source: Ecol Evol. 2023 Apr 10;13(4):e9972. doi: 10.1002/ece3.9972 (PMC10085813; doi:10.1002/ece3.9972)
Supplement: Supplementary file 1 — Appendix S1. [file ECE3-13-e9972-s001.pdf]

Soil Map—Clarke and Oconee Counties, Georgia

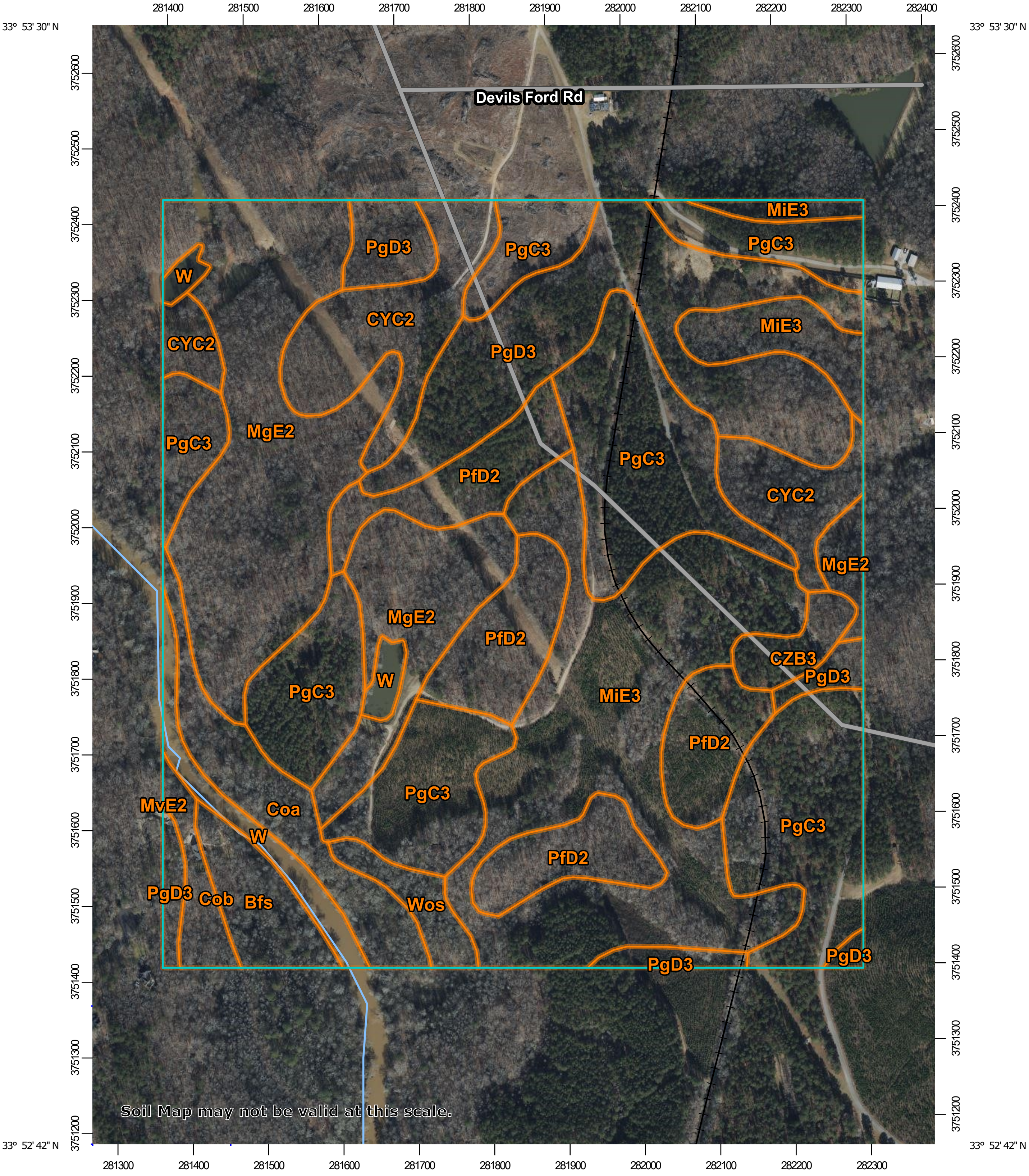

Map Scale: 1:7,210 if printed on A portrait (8.5" x 11") sheet.

N

Meters: 0, 100, 200, 400, 600

Feet: 0, 350, 700, 1400, 2100

Map projection: Web Mercator Corner coordinates: WGS84 Edge tics: UTM Zone 17N WGS84

MAP LEGEND

Area of Interest (AOI)

- Area of Interest (AOI)

Soils

- Soil Map Unit Polygons
- Soil Map Unit Lines
- Soil Map Unit Points

Special Point Features

- Blowout
- Borrow Pit
- Clay Spot
- Closed Depression
- Gravel Pit
- Gravelly Spot
- Landfill
- Lava Flow
- Marsh or swamp
- Mine or Quarry
- Miscellaneous Water
- Perennial Water
- Rock Outcrop
- Saline Spot
- Sandy Spot
- Severely Eroded Spot
- Sinkhole
- Slide or Slip
- Sodic Spot
- Spoil Area
- Stony Spot
- Very Stony Spot
- Wet Spot
- Other
- Special Line Features

Water Features

- Streams and Canals

Transportation

- Rails
- Interstate Highways
- US Routes
- Major Roads
- Local Roads

Background

- Aerial Photography

MAP INFORMATION

The soil surveys that comprise your AOI were mapped at 1:15,800.

Warning: Soil Map may not be valid at this scale.

Enlargement of maps beyond the scale of mapping can cause misunderstanding of the detail of mapping and accuracy of soil line placement. The maps do not show the small areas of contrasting soils that could have been shown at a more detailed scale.

Please rely on the bar scale on each map sheet for map measurements.

Source of Map: Natural Resources Conservation Service
Web Soil Survey URL:
Coordinate System: Web Mercator (EPSG:3857)

Maps from the Web Soil Survey are based on the Web Mercator projection, which preserves direction and shape but distorts distance and area. A projection that preserves area, such as the Albers equal-area conic projection, should be used if more accurate calculations of distance or area are required.

This product is generated from the USDA-NRCS certified data as of the version date(s) listed below.

Soil Survey Area: Clarke and Oconee Counties, Georgia
Survey Area Data: Version 15, Sep 14, 2022

Soil map units are labeled (as space allows) for map scales 1:50,000 or larger.

Date(s) aerial images were photographed: Dec 19, 2020—Dec 27, 2020

The orthophoto or other base map on which the soil lines were compiled and digitized probably differs from the background imagery displayed on these maps. As a result, some minor shifting of map unit boundaries may be evident.

Map Unit Legend

Map Unit Symbol	Map Unit Name	Acres in AOI	Percent of AOI
Bfs	Buncombe loamy sand	5.1	2.2%
Coa	Congaree soils and alluvial land	8.5	3.7%
Cob	Chewacla soils and alluvial land	2.8	1.2%
CYC2	Cecil sandy loam, 6 to 10 percent slopes, moderately eroded	17.4	7.5%
CZB3	Cecil sandy clay loam, 2 to 6 percent slopes, severely eroded	2.7	1.2%
MgE2	Madison sandy loam, 15 to 25 percent slopes, eroded	40.5	17.4%
MiE3	Madison sandy clay loam, 10 to 25 percent slopes, severely eroded	41.7	17.9%
MvE2	Musella clay loam, 15 to 25 percent slopes, eroded	0.0	0.0%
PfD2	Pacolet sandy loam, 10 to 15 percent slopes, moderately eroded	23.0	9.8%
PgC3	Pacolet sandy clay loam, 6 to 10 percent slopes, severely eroded	53.0	22.7%
PgD3	Pacolet sandy clay loam, 10 to 15 percent slopes, severely eroded	30.7	13.1%
W	Water	5.3	2.3%
Wos	Wehadkee and Alluvial land, wet	2.8	1.2%
**Totals for Area of Interest**		**233.5**	**100.0%**
