# Supplementary material for: Spatiotemporal variation in the competitive environment, with implications for how climate change may affect a species with parental care
Source: Ecol Evol. 2023 Apr 10;13(4):e9972. doi: 10.1002/ece3.9972 (PMC10085813; doi:10.1002/ece3.9972)
Supplement: Supplementary file 2 — Appendix S2. [file ECE3-13-e9972-s002.pdf]

Site	Date beetles were collected from trap in paper	No. *N. orbicollis* captured	Revised date range for comparison across papers	Revised No. of *N. orbicollis* to account for missing dates
Whitehall 2022	10-Mar	0	10-Mar	
Whitehall 2022	24-Mar	2	24-Mar	
Kentucky 1968	29-Mar	0	1-Apr	
Whitehall 2022	1-Apr	0	1-Apr	
Kentucky 1968	6-Apr	1	8-Apr	
Whitehall 2022	8-Apr	1	8-Apr	
Ontario 1980	12-Apr	0	15-Apr	
Kentucky 1968	12-Apr	0	15-Apr	
Whitehall 2022	15-Apr	0	15-Apr	
Kentucky 1968	19-Apr	3	22-Apr	
Ontario	19-Apr	0	22-Apr	
Whitehall 2022	22-Apr	6	22-Apr	
Ontario	26-Apr	0	29-Apr	
Kentucky 1968	26-Apr	9	29-Apr	
Whitehall 2022	29-Apr	72	29-Apr	
Kentucky 1968	3-May	9	6-May	
Ontario	6-May	0	6-May	
Whitehall 2022	6-May	37	6-May	
Whitehall 2022	10-May	19	10-May	
Kentucky 1968	12-May	54	10-May	
Ontario 1982	14-May	14	10-May	
Kentucky 1968	17-May	106	19-May	
Whitehall 2022	19-May	61	19-May	
Ontario 1982	21-May	18	19-May	
Kentucky 1968	25-May	91	26-May	
Whitehall 2022	26-May	32	26-May	
Ontario 1982	28-May	1	26-May	
Kentucky 1968	31-May	102	2-Jun	
Whitehall 2022	2-Jun	22	2-Jun	
Ontario 1982	4-Jun	81	2-Jun	
Kentucky 1968	8-Jun	129	9-Jun	
Whitehall 2022	9-Jun	27	9-Jun	
Ontario 1982	11-Jun	315	9-Jun	
Kentucky 1968	15-Jun	77	15-Jun	
Whitehall 2022	15-Jun	27	15-Jun	
Ontario 1982	18-Jun	98	15-Jun	
Whitehall 2022	21-Jun	24	21-Jun	
Ontario 1982	25-Jun	54	21-Jun	
Whitehall 2022	28-Jun	37	28-Jun	
Ontario 1982	2-Jul	181	28-Jun	
Whitehall 2022	6-Jul	49	6-Jul	

Ontario 1982	9-Jul	61	6-Jul	
Whitehall 2022	14-Jul	52	14-Jul	
Ontario 1982	16-Jul	154	14-Jul	
Whitehall 2022	22-Jul	31	22-Jul	
Ontario 1982	23-Jul	66	22-Jul	
Ontario 1982	30-Jul	170	10-Aug	Ontario 1982: 398
Ontario 1982	6-Aug	180	10-Aug	
Ontario 1982	13-Aug	48	10-Aug	
Whitehall 2022	10-Aug	15	10-Aug	
Whitehall 2022	19-Aug	25	19-Aug	
Ontario 1982	20-Aug	51	19-Aug	
Ontario 1982	27-Aug	100	2-Sep	Ontario 1982: 140
Whitehall 2022	2-Sep	40	2-Sep	
Ontario 1982	3-Sep	75	2-Sep	
Ontario 1982	10-Sep	91	23-Sep	Ontario 1982: 134
Ontario 1982	15-Sep	24	23-Sep	
Ontario 1982	22-Sep	19	23-Sep	
Whitehall 2022	23-Sep	39	23-Sep	
Ontario 1982	29-Sep	13	29-Sep	
Whitehall 2022	29-Sep	27	29-Sep	
Kentucky 1968	4-Oct	17	6-Oct	
Ontario 1982	5-Oct	9	6-Oct	
Whitehall 2022	6-Oct	10	6-Oct	
Kentucky 1968	11-Oct	23	13-Oct	
Ontario 1982	13-Oct	1	13-Oct	
Whitehall 2022	13-Oct	13	13-Oct	
Kentucky 1968	18-Oct	21	21-Oct	
Ontario 1982	20-Oct	6	21-Oct	
Whitehall 2022	21-Oct	10	21-Oct	
Kentucky 1968	25-Oct	16	28-Oct	
Ontario 1982	27-Oct	0	28-Oct	
Whitehall 2022	28-Oct	7	28-Oct	
Kentucky 1968	1-Nov	9	4-Nov	
Whitehall 2022	2-Nov	8	4-Nov	
Ontario 1982	3-Nov	6	4-Nov	
Kentucky 1968	8-Nov	2	11-Nov	
Whitehall 2022	11-Nov	10	11-Nov	
Kentucky 1968	15-Nov	0	16-Nov	
Whitehall 2022	16-Nov	2	16-Nov	
Kentucky 1968	22-Nov	0	22-Nov	
Whitehall 2022	22-Nov	0	22-Nov	

Site	year	Date beetles were collected from trap in paper	No. *N. orbicollis* captured	No. *N. tomentosus* captured	Revised date range for comparison across papers	Revised No. of *N. orbicollis* to account for missing dates	Revised No. of *N. tomentosus* to account for missing dates
Whitehall	2002	3-Jan	0	0	3-Jan	0	0
Whitehall	2002	3-Feb	0	0	3-Feb	0	0
Whitehall	2002	10-Feb	0	0	10-Feb	0	0
Whitehall	2002	17-Feb	0	0	17-Feb	0	0
Whitehall	2002	24-Feb	0	0	24-Feb	0	0
Whitehall	2002	3-Mar	0	0	3-Mar	0	0
Whitehall	2002	10-Mar	0	0	10-Mar	0	0
Whitehall	2022	10-Mar	0	0	10-Mar	0	0
Whitehall	2022	24-Mar	2	0	24-Mar	2	0
Whitehall	2002	31-Mar	0	0	1-Apr	0	0
Whitehall	2022	1-Apr	0	0	1-Apr	0	0
Whitehall	2002	7-Apr	5	0	8-Apr	5	0
Whitehall	2022	8-Apr	1	0	8-Apr	1	0
Whitehall	2002	14-Apr	3	0	15-Apr	3	0
Whitehall	2022	15-Apr	0	0	15-Apr	0	0
Whitehall	2002	21-Apr	3	0	22-Apr	3	0
Whitehall	2022	22-Apr	6	0	22-Apr	6	0
Whitehall	2002	28-Apr	19	0	29-Apr	19	0
Whitehall	2022	29-Apr	72	0	29-Apr	72	0
Whitehall	2002	5-May	3	0	6-May	3	0
Whitehall	2022	6-May	37	1	6-May	37	1
Whitehall	2002	12-May	3	0	10-May	3	0
Whitehall	2022	10-May	19	0	10-May	19	0
Whitehall	2002	19-May	4	0	19-May	4	0
Whitehall	2022	19-May	61	1	19-May	61	1
Whitehall	2002	26-May	4	6	26-May	4	6
Whitehall	2022	26-May	32	1	26-May	32	1
Whitehall	2002	3-Jun	2	19	2-Jun	2	19
Whitehall	2022	2-Jun	22	5	2-Jun	22	5

Whitehall	2002	10-Jun	13	23	9-Jun	13	23
Whitehall	2022	9-Jun	27	10	9-Jun	27	10
Whitehall	2002	17-Jun	25	4	15-Jun	25	4
Whitehall	2022	15-Jun	27	2	15-Jun	27	2
Whitehall	2022	21-Jun	24	6	21-Jun	24	6
Whitehall	2022	28-Jun	37	1	28-Jun	37	1
Whitehall	2002	8-Jul	30	1	6-Jul	30	1
Whitehall	2022	6-Jul	49	0	6-Jul	49	0
Whitehall	2002	15-Jul	18	1	14-Jul	18	1
Whitehall	2022	14-Jul	52	0	14-Jul	52	0
Whitehall	2002	22-Jul	50	0	22-Jul	50	0
Whitehall	2022	22-Jul	31	0	22-Jul	31	0
Whitehall	2002	29-Jul	17	1	10-Aug	32	1
Whitehall	2002	5-Aug	15	0	10-Aug		
Whitehall	2022	10-Aug	15	0	10-Aug	15	0
Whitehall	2002	12-Aug	10	0	19-Aug	31	0
Whitehall	2002	19-Aug	21	0	19-Aug		
Whitehall	2022	19-Aug	25	0	19-Aug	25	0
Whitehall	2002	26-Aug	29	0	2-Sep	43	1
Whitehall	2002	2-Sep	14	1	2-Sep		
Whitehall	2022	2-Sep	40	1	2-Sep	40	1
Whitehall	2002	16-Sep	10	4	23-Sep	16	8
Whitehall	2002	23-Sep	6	4	23-Sep		
Whitehall	2022	23-Sep	39	1	23-Sep	39	1
Whitehall	2002	30-Sep	22	23	29-Sep	22	23
Whitehall	2022	29-Sep	27	1	29-Sep	27	1
Whitehall	2002	6-Oct	6	3	6-Oct	6	3
Whitehall	2022	6-Oct	10	2	6-Oct	10	2
Whitehall	2002	14-Oct	11	24	13-Oct	11	24
Whitehall	2022	13-Oct	13	8	13-Oct	13	8
Whitehall	2002	21-Oct	2	12	21-Oct	2	12
Whitehall	2022	21-Oct	10	0	21-Oct	10	0
Whitehall	2022	28-Oct	7	7	28-Oct	7	7

Whitehall	2002	4-Nov	0	3	4-Nov	0	3
Whitehall	2022	2-Nov	8	0	4-Nov	8	0
Whitehall	2002	11-Nov	9	9	11-Nov	9	9
Whitehall	2022	11-Nov	10	6	11-Nov	10	6
Whitehall	2002	18-Nov	0	0	16-Nov	0	0
Whitehall	2022	16-Nov	2	1	16-Nov	2	1
Whitehall	2002	25-Nov	0	5	22-Nov	0	5
Whitehall	2022	22-Nov	0	0	22-Nov	0	0
Whitehall	2002	1-Dec	0	0	1-Dec	0	0
Whitehall	2022	1-Dec	0	8	1-Dec	0	8
Whitehall	2002	9-Dec	0	0	8-Dec	0	0
Whitehall	2022	8-Dec	0	1	8-Dec	0	1
Whitehall	2002	16-Dec	0	0	15-Dec	0	0
Whitehall	2022	15-Dec	0	0	15-Dec	0	0
